# Magnetic Weyl Semimetal in BaCrSe_2_ with Long‐Distance Distribution of Weyl Points

**DOI:** 10.1002/advs.202301474

**Published:** 2023-05-28

**Authors:** Wenli Sun, Bingyang Li, Xiaorong Zou, Runhan Li, Baibiao Huang, Ying Dai, Chengwang Niu

**Affiliations:** ^1^ School of Physics, State Key Laboratory of Crystal Materials Shandong University Jinan 250100 China

**Keywords:** DFT calculations, Fermi arc, magnetic Weyl semimetal, Weyl points

## Abstract

Weyl semimetals (WSMs) have attracted great attentions that provide intriguing platforms for exploring fundamental physical phenomena and future topotronics applications. Despite the fact that numerous WSMs are achieved, WSMs with long‐distance distribution of Weyl points (WPs) in given material candidates remain elusive. Here, the emergence of intrinsic ferromagnetic WSMs in BaCrSe_2_ with the nontrivial nature explicitly confirmed by the Chern number and Fermi arc surface states analysis is theoretically demonstrated. Remarkably, unlike previous WSMs for which opposite chirality WPs are located very close to each other, the WPs of BaCrSe_2_ host a long‐distance distribution, as much as half of the reciprocal space vector, suggesting that the WPs are highly robust and difficult to be annihilated by perturbations. The presented results not only advance the general understanding of magnetic WSMs but also put forward potential applications in topotronics.

## Graphical abstract

Based on first‐principles calculations, the intrinsic Weyl semimetal (WSM) phase in BaCrSe_2_ is theoretically demonstrated. Unlike previous WSMs for which opposite chirality Weyl points (WPs) are located very close to each other, the WPs of BaCrSe_2_ host a long‐distance distribution, suggesting that the WPs are highly robust and difficult to be annihilated by perturbations.

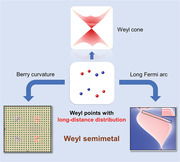

## 1 Introduction

Weyl semimetals (WSMs), which describe the low‐energy excitation of Weyl points (WPs) with isolated double degenerate band crossings near the Fermi level, have stimulated significant interest currently.^[^ ^1^, ^2^, ^3^ ^]^ WPs with positive and negative chirality must exist in pairs on the basis of no‐go theorem,^[^ ^4^ ^]^ and can be regarded as the respective source and drain of Berry curvatures in momentum space.^[^ ^5^, ^6^, ^7^ ^]^ The integral of Berry curvatures of the closed manifold constructed around WPs are the Chern number C ^[^ ^8^ ^]^ and therefore the transition between Chern insulators and ordinary insulators occurs in the 2D planes between WPs, which also makes WSMs have non‐closed Fermi arcs connecting WPs of different chirality on their surface states.^[^ ^9^, ^10^ ^]^. The topological Fermi arc states play important roles in confirming the WSMs as that can be directly observed by angle‐resolved photoemission spectroscopy.^[^ ^11^, ^12^, ^13^, ^14^ ^]^ These peculiarities lead to a series of exotic physical properties in WSMs, such as the chiral anomaly effect,^[^ ^14^ ^]^ high‐mobility carriers,^[^ ^14^, ^15^, ^16^ ^]^ negative magnetoresistive,^[^ ^17^, ^18^, ^19^ ^]^ giant anisotropic optical response,^[^ ^20^ ^]^ and giant intrinsic spin/anomalous Hall effects.^[^ ^21^, ^22^ ^]^

WSMs can be achieved in both the nonmagnetic and magnetic materials with spatial inversion and time‐reversal symmetry breaking, respectively.^[^ ^1^, ^2^, ^3^ ^]^ Merging complex interplay between magnetism and band topology, magnetic WSMs have recently received enormous interests with a variety of striking physical phenomena constantly proposed,^[^ ^22^, ^23^, ^24^, ^25^, ^26^, ^27^ ^]^ such as the minimum number of WPs that only proposed in ferromagnetic (FM) MnBi_2_Te_4_^[^ ^28^, ^29^ ^]^ EuCd_2_As_2_,^[^ ^30^, ^31^, ^32^ ^]^ and K_2_Mn_3_(AsO_4_)_3_.^[^ ^33^ ^]^ Furthermore, the topology can be controlled by tailoring the magnetism, for example, the topological nodal‐line semimetals can be obtained in WSMs C_4_CrSi_3_^[^ ^34^ ^]^ and EuB_6_^[^ ^35^ ^]^ as the magnetization direction is tuned. However, the obtained WPs with opposit chirality lying on both sides of the original nodal‐lines and thus their distance is indeed quite small,^[^ ^34^, ^35^ ^]^ faced as well in nonmagnetic WSMs where WPs are obtained by switching on the spin‐orbit coupling (SOC).^[^ ^6^, ^7^ ^]^ As generally accepted, opposite chirality WPs can be annihilated when they reach the same *K* point, therefore, a longer distance will make the WPs more robust against perturbations.^[^ ^36^ ^]^ In spite of extensive efforts so far, suitable candidates of WSMs with long‐distance distribution of WPs still remains challenging.

In the present work, using first‐principles calculations, we show that BaCrSe_2_ with intrinsic FM ordering is a type‐I WSM. We find four pairs of WPs around the Fermi level in the whole Brillouin zone (BZ) with linear dispersion for the cones. Nonzero Chern numbers C=±1 are indeed achieved through a closed surface that encompasses a distinct WP, and the Berry curvature converging and/or diverging at the opposite chirality WPs. Remarkably, we put forward that, in contrast to previously reported WSMs, robust WPs can emerge in BaCrSe_2_ with a long‐distance distribution, reaching as much as half of the reciprocal space vector. Moreover, long Fermi arcs of the topological surface states connecting the WPs are studied as well. In addition, non‐zero Chern number and $Z_{2}$ topological invariant, C=2 and $Z_{2}=1$, are obtained for planes with different ways of crossing the WPs, further explicitly confirming the nontrival WSM nature of BaCrSe_2_. Our results indicate that BaCrSe_2_ provides a promising candidate for both the research of magnetic WSMs and innovative spintronics applications.

## 2 Methods

Based on density functional theory, the calculations are performed using the Vienna ab initio simulation package.^[^ ^37^, ^38^ ^]^ Perdew–Burke–Ernzerhof of generalized gradient approximation (GGA) is used for the exchange correlation potential.^[^ ^39^ ^]^ SOC is included in the calculations self‐consistently. The cutoff energy is set to 450 eV for the plane‐wave basis and a self‐consistent field computation is performed with a convergence of 10^−6^ per atom. The structures are relaxed until the residual force on each atom is less than 0.01 eV Å^‐1^. The GGA+U method with a value of U = 3.0 eV for Cr‐3*d* electrons is used to correct the Coulomb interaction. The phonon calculations are performed by using the density functional perturbation theory as implemented in the PHONOPY package.^[^ ^40^ ^]^ The maximally located Wannier functions (MLWFs) are constructed using the Wannier90 code.^[^ ^41^, ^42^ ^]^

## 3 Results and Discussion

**Figure 1:**
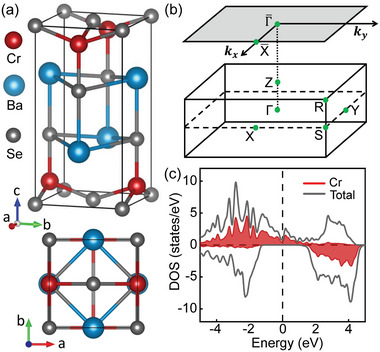
a) Crystal structure of bulk BaCrSe_2_ in space group *P*4/*nmm*. b) Bulk and (001) surface Brillouin zones (BZs) with the corresponding high symmetry points noted as green dots. c) Total and partial density of states for BaCrSe_2_ without spin‐orbit coupling (SOC). The positive and negative values represent the spin‐up and spin‐down states, respectively, showing that the BaCrSe_2_ is in ferromagnetic configuration.

BaCrSe_2_ crystalizes in a tetragonal crystal structure with the space group *P*4/*nmm*,^[^ ^43^, ^44^, ^45^ ^]^ as shown in **Figure** **1**a,b. Each unit cell consists of eight atoms with two Ba in the Wyckoff 2c position, two Cr in the 2c position, and four Se in the 2a,c positions. The optimized lattice parameters of the BaCrSe_2_ are *a*/*b* = 4.84 Å and *c* = 9.55 Å. To check its energetic stability, the formation energy is calculated by $E_{f}=E_{BaCrSe_{2}}-\mu_{Ba}-\mu_{Cr}-2\mu_{Se}$, where $E_{BaCrSe_{2}}$ is the total energy of the BaCrSe_2_, and µ_*Ba*_, µ_*Cr*_, and µ_*Se*_ are the chemical potentials of Ba, Cr, and Se atoms, respectively. The calculated formation energy of ‐3.082 eV indicates that the BaCrSe_2_ is energetically stable. To confirm the dynamical stability of BaCrSe_2_, the phonon band structures are calculated and all phonon branches are positive in the entire BZ, indicating that the BaCrSe_2_ is dynamically stable and difficult to destroy once formed (Figure S1, Supporting Information).^[^ ^46^ ^]^ The bulk and (001) surface BZs are shown in Figure 1b with the high‐symmetry points noted on.

The calculated magnetic moments on each Cr of BaCrSe_2_ are about 4 µ_*B*_ deriving from Cr^2 +^ with a 3*d* ^4^ configuration, and it leads to an intrinsic FM ground state based on the foundational Goodenough–Kanamori rules due to the Cr‐Se‐Cr bond angle approaching 90^o^.^[^ ^47^, ^48^ ^]^ It can be further verified by the calculation of magnetization energy with *E*_*mag*_ = *E*_*FM*_ − *E*_*AFM*_, where *E*_*FM*_ and *E*_*AFM*_ represent the total energies of the system under FM and antiferromagnetic (AFM) configurations, respectively, and the FM ground state is energetically favored by about 89 meV than AFM configuration. Taking SOC into consideration, we have carefully checked the spin orientations for both the out‐of‐plane and in‐plane directions, and the magnetic anisotropy energy *E*_MAE_ is calculated to be 0.05 meV with the in‐plane direction lower in energy than the out‐of‐plane case.

**Figure 2:**
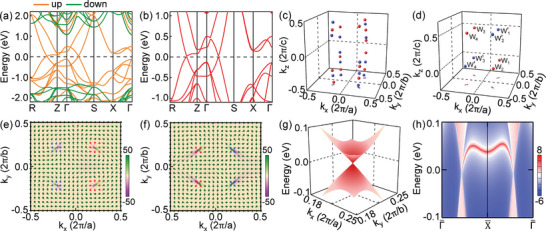
Band structures of bulk BaCrSe_2_ a) without and b) with SOC. Location of Weyl points (WPs) in the bulk BZ c) without and d) with SOC. The spheres and underside dots represent the WPs in 3D bulk BZ and their projection on the (001) surface BZ, respectively. The distributions of Berry curvature $\Omega_{xy}^{kk}$ for the planes e) k_*z*_ = 0 and f) k_*z*_ = 0.5. The arrows represent the in‐plane direction of the Berry curvature, revealing the long‐distance distribution of opposite chirality WPs. g) 3D band structure around the WP W_1_ as marked in (d). h) Surface states on the (010) surface of BaCrSe_2_.

Figure 1c presents the total density of states (DOS) and partial DOS of Cr atoms under the FM ground state in the absence of SOC. As expected, they are clearly spin‐polarized. A few DOS around the Fermi level indicate that the BaCrSe_2_ is not an insulator and host simple band electronic structures over there, as is further verified by the band structures illustrated in **Figure** **2**a, in which the spin‐down subbands emerge a giant band gap but the spin‐up subbands show the metallic property with exotic band crossings emerging along the Γ‐S and R‐Z paths. Then we search for the WPs from the whole BZ and gain 14 pairs of WPs around the Fermi level, as shown in Figure 2c. Their projections on the (001) surface BZ concentrate on 4 points along the diagonal direction, suggesting that the WPs distribute in four curves roughly parallel to the k_*z*_ direction.

In the presence of SOC, as shown in Figure 2b, the band structures do not change significantly along the high symmetric paths. Interestingly, one notes that the SOC leads to the band gap opening for many of the WPs, that is, the coressponding WP disappers, and four pairs of symmetrical WPs survive around the Fermi level. Their momentum space locations in fractional coordinates are (±0.214, ±0.214, 0) and (±0.191, ±0.191, 0.5), respectively, as displayed in Figure 2d. Moreover, from the 3D band structures shown in Figure 2g, one can see clearly that there is a distinct band point touching with linearly dispersed at the WP, that is, a point‐like Fermi surface, and thus the BaCrSe_2_ is a type‐I WSM.^[^ ^49^ ^]^ Remarkably, unlike the previously proposed WSMs, the distance between WPs is very far, reaching as much as half of the reciprocal space vector, suggesting that the WPs of BaCrSe_2_ are highly robust and difficult to be annihilated by perturbations. This is clearly confirmed by introducing various perturbations, such as the changes of U, displacement of Fermi energy, and the symmetry change. Where, although the band structures change slightly, the WPs with long‐distance distribution remain intact (Figures S3–S5, Supporting Information).^[^ ^46^ ^]^

To firmly confirm the chirality of the WPs as well as their topological nature, we then focus on the Berry curvatures, given by Ωijkk=2Im∑nocc⟨∂kiukn|∂kjukn⟩ (*i*/*j* = *x*, *y*, and *z*).^[^ ^50^ ^]^ Integral of Berry curvatures of any closed surface is the sum of WPs included in the closed surface, in which the integral symbol is the sign of the “magnetic charge” corresponding to the WPs, and if the closed surface contains only one WP, the integral value is called as the chirality of that WP.^[^ ^1^, ^2^ ^]^ Thus, we take the 2D spherical closed surface with a very small radius centered on the WPs and integrate the Berry curvatures to obtain the chirality of the WPs. As illustrated in Figure 2c,d, where red and blue dots are used to represent the positive and negative chirality, respectively, the same number of WPs for opposite chiralities makes the Berry curvature integral to the entire boundary plane of the BZ to zero, revealing that the WPs always appear in pairs with opposite chirality. To show precisely the chirality and location of the WPs, we plot the Berry curvatures of planes k_*z*_ = 0 and k_*z*_= 0.5 in Figure 2e,f, respectively. Clearly, one can see that the dominant amplitude of the Berry curvature $\Omega_{ij}^{kk}$ is distributed mainly around the WPs, and the $\Omega_{xy}^{kk}$ diverge at the WPs with positive chirality while converge at the WPs with negative chirality. The adjacent WPs with opposite chirality, *W*_*i*_ and $W_{i}^{\prime }$, lying on k_*z*_ = 0 and k_*z*_= 0.5 planes, respectively, with a distance of 0.33 Å^−1^ that is 50% of the inverse lattice constant, much larger than the previous ones.^[^ ^6^, ^7^ ^]^

**Figure 3:**
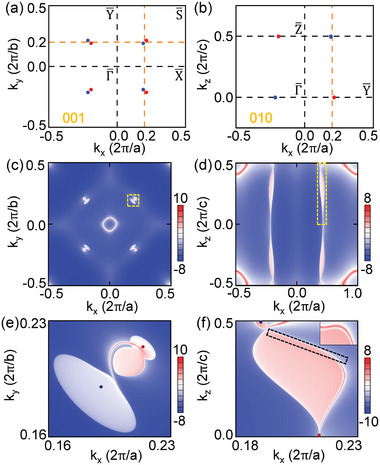
Projections of bulk WPs on the a) (001) and b) (010) surface BZs in the presence of SOC. Fermi‐arc surface states on c) (001) and d) (010) surfaces, and the corresponding zoom‐in views are shown in (e,f), respectively.

A distinctive feature of WSM is the surface Fermi arc connected to the opposite WPs in the surface BZ.^[^ ^9^ ^]^ Taking SOC into consideration, projections of the eight WPs in the surface BZs along (001) and (010) directions are displayed in **Figure** **3**a,b, respectively. We find that all of the eight WPs project on different points in the (001) surface BZ, while, in the (010) surface BZ, two WPs with the same chirality project on the same point. Thus, the appearance of Fermi arcs on both the (001) and (010) surfaces are guaranteed. The surface density of states for BaCrSe_2_ are computed by the tight‐binding Hamiltonian generated by the MLWFs of bulk BaCrSe_2_. Figure 2h presents the surface states on the (010) surface. It is metallic with the surface projections of bulk WPs clearly visible, and remarkably the nontrivial surface states connect the projections. Topological Fermi arc surface states are displayed in Figure 3c,d for the (001) and (010) surfaces, respectively, and their marked regions are zoomed‐in in Figure 3e,f. Both the number and pattern of the Fermi arcs are different, just as expected. For the (001) surface, the WPs project on different points, one Fermi arc connects each pair of the opposite chirality WPs, $W_{i}$_*i*_ and $W_{i}^{\prime }$. However, for the (010) surface, two Fermi arcs connect to each of the projection points, because they project in pairs with the same chiral WPs as discussed above.

**Figure 4:**
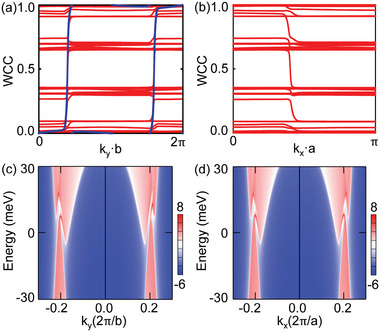
Evolution of the wannier charge centers (WCCs) over all occupied bands for a) k_*x*_ = 0.2 plane and b) k_*y*_ = 0.2 plane, suggesting the nontrivial characters with C=2 and $Z_{2}=1$, respectively. Surface states on (001) surface along c) k_*x*_ = 0.2 and d) k_*y*_ = 0.2.

In 3D BZ of BaCrSe_2_, 2D planes will undergo a band gap closing and reopening process when crossing the WPs with the change of plane coordinates, which naturally have rich topological phenomena and phase transitions. We select four planes k_*x*_/k_*y*_ = 0/0.2 for comparison, and their relative positions with the WPs are illustrated in Figure 3a. In fact, the k_*x*_= 0 and k_*y*_ = 0 planes have the similar situations, both of them are far from the WPs. Four pairs of WPs are evenly distributed on both sides of them, and, on each side, the total chirality of WPs is 0, resulting in a trivial insulator phase for both k_*x*_ = 0 and k_*y*_ = 0 planes (Figure S2, Supporting Information).^[^ ^46^ ^]^ On the other hand, the k_*x*_ = 0.2 and k_*y*_ = 0.2 planes divide two pairs of nearby WPs evenly on both sides of the planes, that is, two WPs on each side. However, the total chirality of WPs on two sides is different. The positive and negative WPs localize on different sides of the k_*x*_ = 0.2 plane, while there is one positive and one negative WPs on each side of the k_*y*_ = 0.2 plane. It is, therefore, the total chirality of WPs is 2 (or ‐2) on one side of k_*x*_ = 0.2 plane, and thus the Chern number is C=2 for k_*x*_ = 0.2 plane, as illustrated in **Figure** **4**a. This is further explicitly confirmed by the calculated edge states plotted in Figure 4c. Although the global band gap is negative, there are two codirectional edge states connecting the “conduction band and valence band” as in other quantum anomalous Hall insulators. Moreover, for k_*y*_ = 0.2 plane, a nontrivial $Z_{2}$ topological invariant is well defined. As shown in Figure 4b, the evolution of wannier charge centers indicates the topological property with $Z_{2}=1$. This is in direct agreement with the emergence of a pair of edge states as shown in Figure 4d.

## 4 Conclusion

In summary, we have demonstrated that FM BaCrSe_2_ is a promising material candidate for the type‐I magnetic WSM with four pairs of WPs around the Fermi level. Moreover, we uncovered that, unlike previous proposed WSMs, the WPs of BaCrSe_2_ are highly robust because the distance between opposite chirality WPs is as far as half of the reciprocal space vector. Our results greatly enrich the prominent topological phenomena and expand the domain of intrinsic magnetic WSMs with highly robust WPs, which are expected to draw great attentions in experiments.

## Conflict of Interest

The authors declare no conflict of interest.

## Supporting information

Supporting Information

## Data Availability

The data that support the findings of this study are available from the corresponding author upon reasonable request.
