# Supplementary material for: Magnetic Weyl Semimetal in BaCrSe2 with Long‐Distance Distribution of Weyl Points
Source: Adv Sci (Weinh). 2023 May 28;10(22):2301474. doi: 10.1002/advs.202301474 (PMC10401091; doi:10.1002/advs.202301474)
Supplement: Supplementary file 1 — Supporting Information [file ADVS-10-2301474-s001.pdf]

ADVANCED SCIENCE
Open Access

Supporting Information

for *Adv. Sci.*, DOI 10.1002/advs.202301474

Magnetic Weyl Semimetal in $BaCrSe_2$ with Long-Distance Distribution of Weyl Points

Wenli Sun, Bingyang Li, Xiaorong Zou, Runhan Li, Baibiao Huang, Ying Dai and Chengwang Niu**

ADVANCED SCIENCE
Open Access

Supporting Information

for advs.202301474

Magnetic Weyl semimetal in $BaCrSe_2$ with long-distance distribution of Weyl points

Wenli Sun, Bingyang Li, Xiaorong Zou, Runhan Li, Baibiao Huang, Ying Dai, and Chengwang Niu

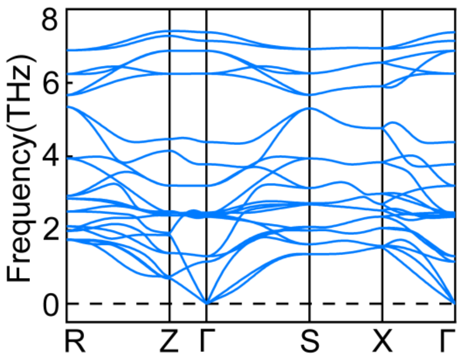

Figure S1. Phonon dispersion of $BaCrSe_2$, showing the system is dynamically stable.

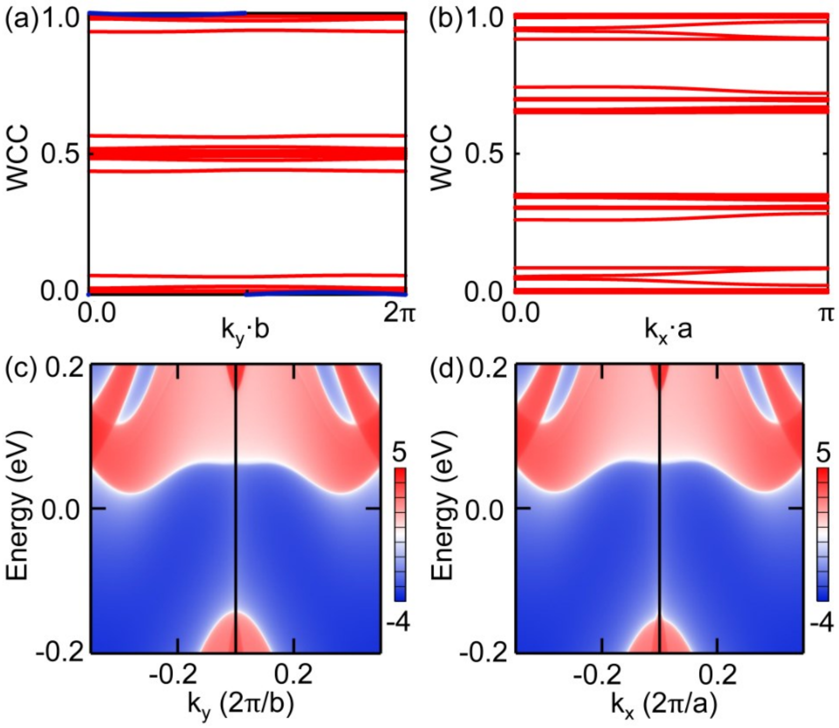

Figure S2. (a) Evolution of the WCC over all occupied bands for (a) $k_x = 0$ plane and (b) $k_y = 0$ plane, suggesting the trivial characters with $C = 0$ and $Z_2 = 0$, respectively. Surface states on (010) surface along (c) $k_x = 0$ and (d) $k_y = 0$.

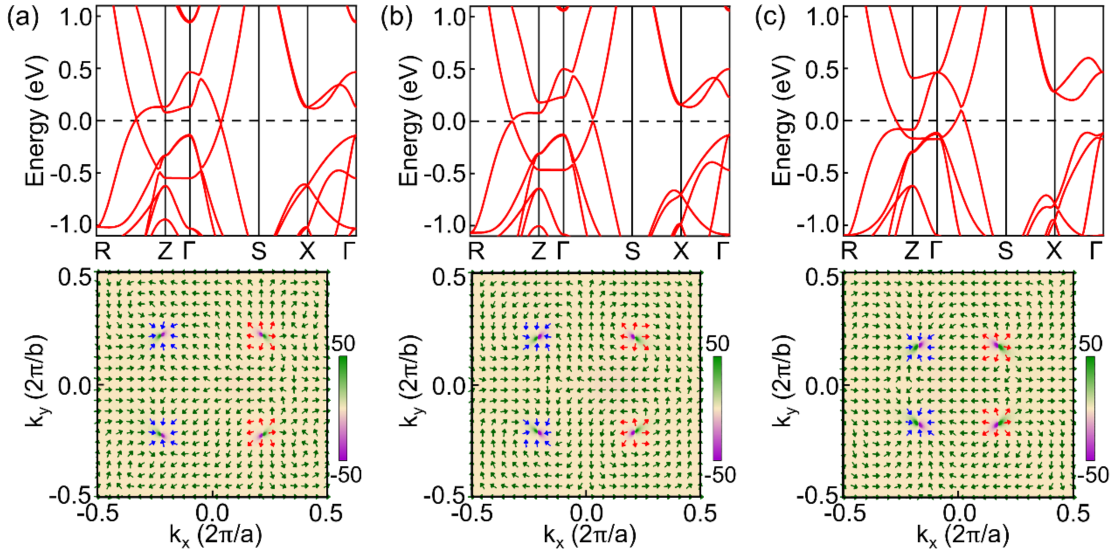

Figure S3. Band structures (above) and the distributions of Berry curvature Ω_{xy}^{kk} for $k_z = 0$ plane (below) of $BaCrSe_2$ with SOC for different Hubbard interaction (a) U = 2 eV, (b) U = 3 eV, and (c) U = 4 eV. The Weyl points are robust to different values of U.

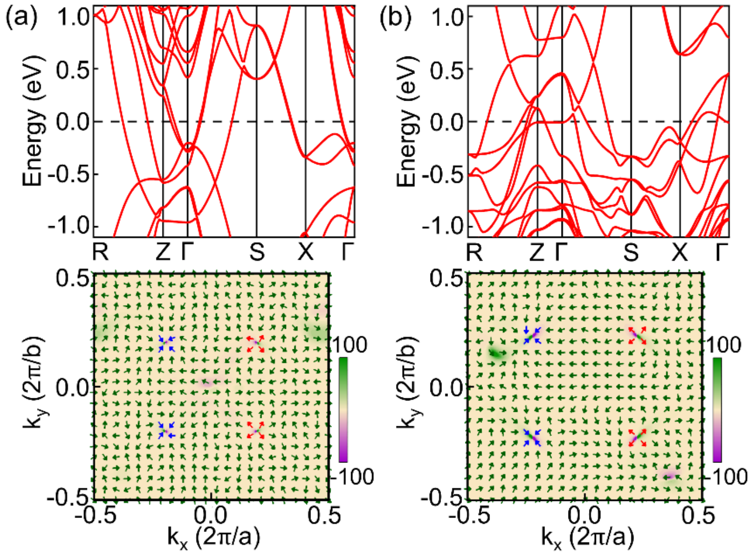

Figure S4. Band structures (above) and the distributions of Berry curvature Ω_{xy}^{kk} for $k_z = 0$ plane (below) of $BaCrSe_2$ with SOC when (a) adding or (b) subtracting an electron to the system. The Weyl points are robust to the displacement of Fermi energy due to the changes in the electron number of occupied states.

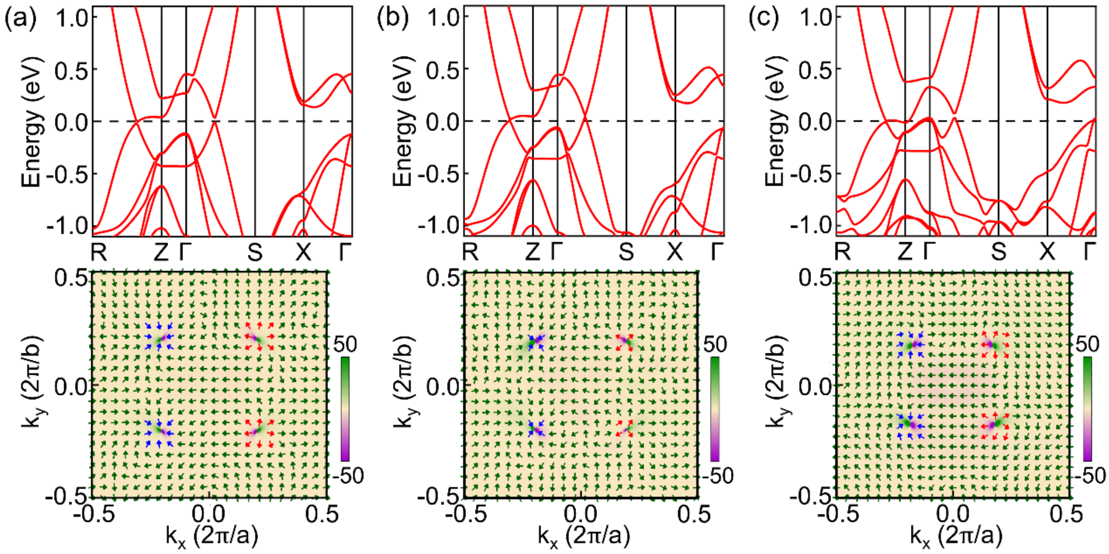

Figure S5. Band structures (above) and the distributions of Berry curvature Ω_{xy}^{kk} for $k_z = 0$ plane (below) of $BaCrSe_2$ with SOC when a Se atom is shifted up (a) 0.1 Å, (b) 0.2 Å and (c) 0.5 Å. The Weyl points are robust to the symmetry change caused by the movement of this atom.
